# Mitochondrial Dysfunction: A Key Player in Brain Aging and Diseases

**DOI:** 10.3390/cimb46030130

**Published:** 2024-03-02

**Authors:** Sydney Bartman, Giuseppe Coppotelli, Jaime M. Ross

**Affiliations:** 1 George and Anne Ryan Institute for Neuroscience, University of Rhode Island, Kingston, RI 02881, USA; 2 Department of Biomedical and Pharmaceutical Sciences, College of Pharmacy, University of Rhode Island, Kingston, RI 02881, USA

**Keywords:** mitochondria, mitochondrial dysfunction, aging, neurodegenerative diseases

## Abstract

Mitochondria are thought to have become incorporated within the eukaryotic cell approximately 2 billion years ago and play a role in a variety of cellular processes, such as energy production, calcium buffering and homeostasis, steroid synthesis, cell growth, and apoptosis, as well as inflammation and ROS production. Considering that mitochondria are involved in a multitude of cellular processes, mitochondrial dysfunction has been shown to play a role within several age-related diseases, including cancers, diabetes (type 2), and neurodegenerative diseases, although the underlying mechanisms are not entirely understood. The significant increase in lifespan and increased incidence of age-related diseases over recent decades has confirmed the necessity to understand the mechanisms by which mitochondrial dysfunction impacts the process of aging and age-related diseases. In this review, we will offer a brief overview of mitochondria, along with structure and function of this important organelle. We will then discuss the cause and consequence of mitochondrial dysfunction in the aging process, with a particular focus on its role in inflammation, cognitive decline, and neurodegenerative diseases, such as Huntington’s disease, Parkinson’s disease, and Alzheimer’s disease. We will offer insight into therapies and interventions currently used to preserve or restore mitochondrial functioning during aging and neurodegeneration.

## 1. Introduction

Recent decades have witnessed a dramatic increase in human longevity due to a combination of factors including broader access to health care, better medical treatments, hygiene, and nutrition [1]. As the lifespan of the human population is increasing, so is the incidence of degenerative diseases with neurodegenerative disorders at the forefront of all forms of chronic disease [2]. An unhealthy aged population will have catastrophic social and financial impacts on our society, considering the cost of long-term and hospice care together with the unpaid care provided by families, if therapeutic interventions to increase healthspan and reduce age-related diseases will not be found soon. Therefore, the necessity to understand the underlying molecular mechanisms of the aging process, particularly brain aging, is imperative.

Aging can be defined as a degenerative process that results from an accumulation of damage to macromolecules, organelles, and cells, leading to organ and tissue dysfunction, and ultimately death [3]. Aging can be thought of as both time-dependent (gradual) and progressive, with damage impairing the functioning of the organism and increasing its vulnerability to environmental challenge and disease [4]. Notably, aging was once seen as an inevitable process; however, a better understanding of the overall process has led to the recognition that aging is malleable and is influenced by both environmental and genetic factors [5]. Early observations on its malleability come from the work of McCay and colleagues in the 1930′s when they discovered that rats on a caloric-restricted diet exhibited prolonged lifespan as compared to those fed ad libitum [6]. Several decades after those initial observations, Kenyon and colleagues in the 1990’s discovered that a mutation in a single gene, *daf-2*, could affect both life and healthspan in *C. elegans*, thus suggesting that aging is not just the mere result of stochastic events, but that it also has a genomic heritable component [7]. Considering that different lifespans are seen amongst various species, it has been predicted that the rate of aging does, in fact, evolve [8]. Within the human population, aging phenotypes are also notably inconsistent between individuals, thereby increasing our obligation to truly understand human aging in the biological context of disease and longevity [9,10].

**Figure 1:**
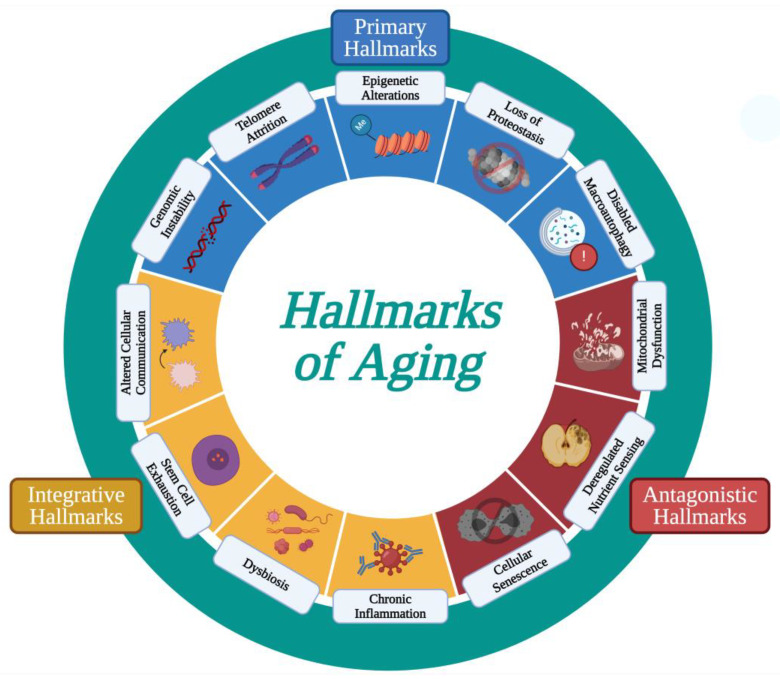
Hallmarks of aging. The hallmarks of aging are categorized into three main groups: Primary Hallmarks (genomic instability, telomere attrition, epigenetic alterations, loss of proteostasis, and disabled macroautophagy), Antagonistic Hallmarks (mitochondrial dysfunction, deregulated nutrient sensing, and cellular senescence), and Integrative Hallmarks (altered intercellular communication, stem cell exhaustion, dysbiosis, and chronic inflammation). Created with BioRender.com accessed on 27 February 2024 and adapted from [12].

In 2013, Carlos López-Otín and colleagues categorized nine “common denominators” of aging, as presented across multiple organisms, to explain the various phenotypes seen within human aging [11]. These are now known as the “hallmarks of aging” and are considered to contribute to the aging process and determine the aging phenotype of an organism when combined. To better explain the complexity of the aging process, López-Otín and colleagues expanded these hallmarks of aging a decade later to now include twelve processes [12]. The twelve hallmarks include the following: genomic instability, telomere attrition, epigenetic alterations, a loss of proteostasis, disabled macroautophagy, mitochondrial dysfunction, deregulated nutrient sensing, cellular senescence, chronic inflammation, dysbiosis, stem cell exhaustion, and altered intercellular communication (Figure 1). The hallmarks have further been grouped into three categories: primary, antagonistic, and integrative. The primary hallmarks (genomic instability, telomere loss, epigenetic alterations, a loss of proteostasis, and disabled macroautophagy) serve as the underlying cause of molecular damage during aging. The antagonistic hallmarks (deregulated nutrient-sensing, mitochondrial dysfunction, and cellular senescence) can be beneficial or deleterious to the organism depending on intensity. Lastly, the integrative hallmarks (chronic inflammation, dysbiosis, stem cell exhaustion, and altered intercellular communication) are those that arise when cellular homeostasis fails to counteract accumulated damage [11,13].

Mitochondrial dysfunction is one of the primary hallmarks of aging and is implicated in several age-related diseases, including forms of cancer, diabetes (type 2), and neurodegenerative diseases, and is a central piece of one of the most popular theories of aging: “The Mitochondrial Theory of Aging”, first postulated by Denham Harman 50 years ago [14,15]. The Mitochondrial Theory of Aging, sometimes also referred to as the Mitochondrial Free-Radical Theory of Aging (MFRTA), is an extension of the Free Radical or Oxidative Stress Theory of Aging, originally proposed in 1956 by Harman, which postulates that aging is the result of accumulative damage caused by free radicals generated by metabolic reactions or as by-products of cellular processes [16]. Harman modified his Free Radical Theory of Aging to incorporate mitochondria in 1972, after several studies pointed to mitochondria as the main place where free radicals are generated [17]. Although the Mitochondrial Theory of Aging has received many criticisms over the years due to contradictory findings, particularly surrounding the role of reactive oxygen species (ROS), it still remarks the central role that mitochondria have in aging research and degenerative diseases [18]. 

Here, we will offer a brief overview of the mitochondrial structure and functions to then discuss the cause of mitochondrial dysfunction in aging and its consequences, with a particular focus on age-related cognitive decline and neurodegenerative diseases. Current therapies and interventions to preserve or restore mitochondrial function during aging and disease states will be discussed as well.

## 2. Mitochondria

**Figure 2:**
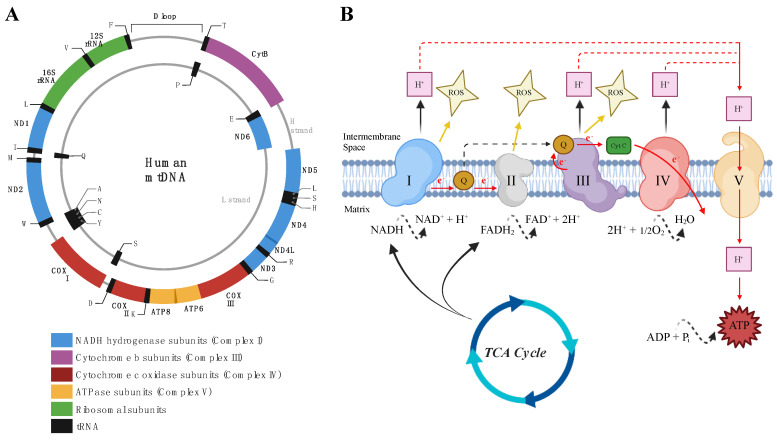
Mitochondrial DNA and oxidative phosphorylation. (**A**) Mitochondrial DNA (mtDNA) is a circular molecule that is organized into individual nucleoids. Human mtDNA is about 16.6 kb and encodes for 22 tRNA genes, 2 rRNA genes, and 13 proteins, which comprise components of the electron transport chain (ETC). The mtDNA encodes proteins for seven subunits of Complex I (blue), one subunit of Complex III (purple), three subunits of Complex IV (red), and two subunits of Complex V (yellow). (**B**) ATP production results from oxidative phosphorylation via Complexes I–IV and Complex V [ATP synthase], with quinone [coenzyme Q] and heme containing protein [cytochrome *c*] as reducing equivalents within the inner mitochondrial membrane. NADH and FADH_2_, produced via the tricarboxylic acid cycle (TCA) or β-oxidation, enter the ETC through Complex I or II. Electrons are then transferred to coenzyme Q and Complex III. Cytochrome *c* acts as a bridge to transport electrons from Complex III and IV. Electrons received by Complex IV are transferred to molecular oxygen to form H_2_O and protons are transferred through the IMM by Complexes I, III, and IV, resulting in the formation of an electrochemical gradient across the IMM, which is utilized by Complex V to produce ATP. Created with BioRender.com accessed on 14 February 2024.

The mitochondrion is the main energy producing organelle in eukaryotic cells and is thought to have become incorporated within the cell approximately 2 billion years ago when a phototrophic α-proteobacterium was engulfed by a familial eukaryotic cell [19]. This symbiotic event led to a derived form of the eukaryotic cell and is believed to have established complex life [20]. Mitochondria play a central role within eukaryotic cells by regulating many processes, such as energy production [21], calcium buffering and homeostasis [22], steroid synthesis [23], cell growth [24], and apoptosis [25], as well as inflammation and ROS production [26,27,28]. The mitochondrial structure resembles its bacterial ancestor with an inner mitochondrial membrane (IMM) and an outer mitochondrial membrane (OMM) surrounding the intermembrane space and matrix compartments [28,29]. Mitochondria also contain their own circular genome known as mitochondrial DNA (mtDNA), which is organized into individual nucleoids comprised of DNA–protein complexes distributed within the matrix [28]. Human mtDNA is about 16.6 kb and encodes for twenty-two tRNA genes, two rRNA genes, and thirteen proteins, which comprise components of the electron transport chain [29] (Figure 2A). Specifically, the mtDNA encodes proteins for seven subunits of the mitochondrial respiratory chain Complex I, one subunit of Complex III, three subunits of Complex IV, and two subunits of Complex V (ATP Synthase). The mitochondrial genome is thought to be a remnant of its ancestral prokaryotic genome, as a majority of genomic information has been transferred to the eukaryotic nucleus or lost throughout evolutionary time [30]. While most of the genetic information (>99%) required for mitochondrial structure and function is found in the nuclear DNA (nDNA) of the eukaryotic cell, the coordinated action of both nuclear and mitochondrial genomes is required during mitochondrial biogenesis [31,32]. Interestingly, mtDNA replicates independently from nDNA and the cell cycle [33]. Energy (ATP) is produced during oxidative phosphorylation (OXPHOS) via five multi-enzyme complexes (Complexes I–IV and ATP Synthase) and two electron carriers (quinone [coenzyme Q] and heme containing protein [cytochrome *c*]), all within the inner mitochondrial membrane [34] (Figure 2B). The reducing equivalents (NADH and FADH_2_) that are generated during glycolysis, the tricarboxylic acid cycle (TCA), or β-oxidation enter into the electron transport chain (ETC) via either Complex I or Complex II and cause electrons to be transferred to coenzyme Q and subsequently to Complex III. Cytochrome *c,* the second electron carrier, acts as a bridge in the transport of electrons from Complex III to IV. Electrons received by Complex IV are then finally transferred to molecular oxygen to form H_2_O [35]. During this process, protons (H^+^) are transferred from the matrix through the IMM by Complexes I, III, and IV, generating an electrochemical gradient across the IMM that is used to power ATP production by Complex V (ATP synthase) [32].

Another function of mitochondria is to regulate intracellular calcium (Ca^2+^) levels, which govern many biological processes, including neuronal excitability [36]. Intracellular Ca^2+^ concentration depends on the extracellular influx through cation channels present on the plasma membrane and on the accumulation and release of Ca^2+^ from intracellular compartments that can accumulate calcium, mainly the endoplasmic reticulum (ER) and mitochondria [37]. Under several circumstances, Ca^2+^ can enter the mitochondrial matrix through the mitochondrial calcium uniporter (MCU), a selective channel present on the IMM that uses the mitochondrial electrochemical potential gradient as a force. Once in the matrix, Ca^2+^ can upregulate the TCA cycle by activating critical TCA cycle enzymes, thus linking Ca^2+^ levels to metabolism [38]. Excitatory cells, such as neurons and muscle cells, possess a second class of calcium channels within the OMM known as voltage-dependent anion channels (VDACs) [39]. VDACs are normally in closed conformation, but spikes in Ca^2+^ concentrations can induce and maintain an “open” confirmation allowing for dissipation of cytoplasmic Ca^2+^. Once accumulated in the matrix, Ca^2+^ will then slowly return to the cytoplasm through the activation of selective Na^+^/Ca^2+^ exchangers [40].

Apoptosis, a programmed cell death orchestrated by caspases, is also regulated by mitochondria through the release of cytochrome *c* from the IMM [41]. Once in the cytoplasm, cytochrome *c* binds to the apoptotic protease activating factor-1 (APAF-1) and forms the ring-like apoptosome complex, which induces apoptosis through the intrinsic pathway by activating caspase-9 [42]. Mitochondria also have a central role in many biosynthetic pathways by providing building blocks for nucleotide synthesis and cholesterol and hormone synthesis, as well as amino acid and heme synthesis [43]. This brief list of cellular processes overseen by mitochondria points to their importance in maintaining the cellular homeostasis of the organism and highlights the impact that mitochondria can have as they become dysfunctional, as implicated in aging and age-related diseases.

## 3. Mitochondrial Dysfunction in Aging: Causes

Swollen, fragmented, and dysfunctional mitochondria have been found in aged tissues from many organisms such as worms (*C. elegans*) [44], flies (*D. melanogaster)* [45], rats [46], mice, and humans [47]. Despite these findings advocating for a conserved mechanism leading to mitochondrial dysfunction, emerging evidence on the contrary suggests that the causes of mitochondrial dysfunction are actually multiple, tissue dependent, and interconnected in such an intricate way that it is rather difficult to identify which pathway could be the primary cause and which one the direct result of mitochondrial dysfunction. 

**Figure 3:**
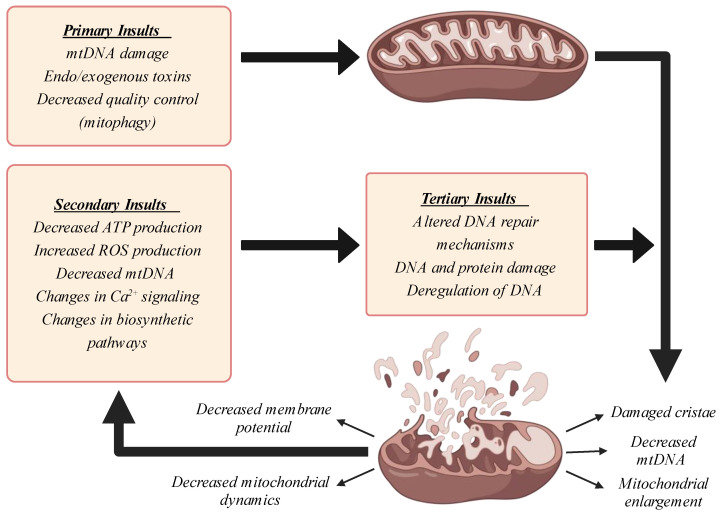
Biological insults of mitochondrial dysfunction. Primary insults can lead to mitochondrial dysfunction in normal functioning cells, which can then induce physiological and functional changes within mitochondria and cause secondary and tertiary insults. Tertiary impairments may also exacerbate mitochondrial impairments or directly induce dysfunction of healthy mitochondria, forming a cycle of mitochondrial damage. Created with BioRender.com accessed on 14 February 2024.

Alongside water, reactive oxygen species (ROS) are a byproduct of the OXPHOS process, and are generated mainly through Complexes I, II, and III [48]. Free radicals, such as hydrogen peroxide (H_2_O_2_), superoxide radicals (O_2_^•−^), and hydroxyl radicals (HO^•^), have been shown to be important secondary messengers; however, due to their extreme reactivity, excessive amounts can damage cellular components, such as lipids and proteins [49]. ROS can directly damage mitochondrial structures that are in proximity to their source of origin, including mtDNA, lipids, and proteins, thus compromising their integrity and inducing mitochondrial dysfunction [49] (Figure 3). For this reason, there is an extensive network of non-enzymatic and enzymatic antioxidant defenses within the cell to ensure healthy ROS levels, such as superoxide dismutase (SOD), catalase (CAT), and glutathione peroxidase (GPx) [50]. Oxidative stress has been shown to induce lipid peroxidation, which can compromise cellular compartmentalization by affecting membrane stability and results in cellular death [51]. Among the several different products that can be formed during lipid peroxidation, 4-hydroxynonenal (4-HNE) has been shown to be the most toxic [52] and increases significantly with aging [53]. Furthermore, proteins can be damaged by ROS and lose their enzymatic activity, conformational state, and stability, which can feed back and increase ROS production by affecting OXPHOS integrity and thus further aggravate mitochondrial dysfunction [54]. ROS species can also interact with nDNA, introducing nDNA mutations by inducing 8-oxo-2′-deoxyguanosine (8-OHdG) formation and compromising the epigenome via CpG methylated isles, thus interfering with the expression of nuclear genes, including the components of mitochondrial complexes or the mitochondrial quality control network, which in turn can affect mitochondrial function [55]. 

Although the role of mtDNA mutations as a primary driver of the aging process remains controversial, there is much agreement that tissue-specific accumulation of somatic mtDNA mutations due to clonal expansion during aging can result in mitochondrial dysfunction [56]. The mitochondrial genome accumulates mutations at a higher rate as compared to the nuclear genome due to several reasons, such as less efficient DNA repair mechanisms, a lack of histones, and exposure to ROS, as well as higher replication cycles [57]. Direct evidence that the accumulation of mtDNA mutations and deletions can lead to aging phenotypes comes from the mtDNA mutator mouse [58,59]. These knock-in mice carry a point mutation (D257A) in the nuclear-encoded mtDNA polymerase-γ (*PolgA*) that abolishes the proofreading ability of the holoenzyme, resulting in high levels of point mutations and deletions in the mitochondrial genome that accumulate over time [58,59]. The mtDNA mutator mouse recapitulates several aspects of the aging phenotype including alopecia (hair loss), graying of the hair, osteoporosis, kyphosis (curvature of the spine), enlarged heart and spleen, anemia, loss of subcutaneous fat, decreased fertility, hearing loss, sarcopenia (muscle loss), decreased body weight and size, and decreased lifespan [58,59,60,61]. The mtDNA mutation load, however, in normally aged mice and in aged human tissues is typically much lower as compared to that in the mtDNA mutator mouse, and is considered to be under the threshold needed to compromise mitochondrial function [62,63,64]. Indeed, eukaryotic cells typically contain several copies of mtDNA molecules (1000–10,000), and mtDNA mutations lead to a condition called heteroplasmy, where normal and mutant mtDNA molecules coexist within the same cell. Thus, it is thought that the number of mutated molecules must reach a certain percentage, which for some cells could be around 80–90% of total mtDNA molecules, for a mutation to exert its deleterious effect [65]. Nonetheless, elevated levels of mtDNA mutations and deletions have been found in normally aged monkeys, rodents, and humans [64,66,67,68], and it has been demonstrated that the amount of mtDNA mutations can affect the onset of aging phenotypes, lifespan, fertility and fecundity, as well as the incidence of brain malformations [61,69]. 

Mitochondrial biogenesis, the mechanism through which new mitochondria are generated within a cell, decreases during aging and is another cause for mitochondrial dysfunction. The regulation of mitochondrial biogenesis is complex and involves many transcriptional factors, thus, the ultimate cause of its decline is not clear [70]. One of the proposed mechanisms is an age-related decrease in the level of the peroxisome proliferator-activated receptor-γ coactivator (PGC)-1α, the master regulator of mitochondrial biogenesis [71]. In this regard, the overexpression of PGC-1α in the muscle of old mice has been shown to restore a subset of young-like molecular patterns [72]. Additionally, increased expression of PGC-1α in mtDNA mutator mice did increase mitochondrial biogenesis and function of skeletal and cardiac muscle, but was unable to revert the mtDNA mutation load [73]. 

Mitochondria comprise a highly dynamic network and undergo frequent fusion and fission events to maintain organelle growth, shape, distribution, and function via lipid membrane and matrix content exchange as well as by isolating dysfunctional branches of its network for removal through mitophagy, a specific form of macro-autophagy [74,75]. The machinery that regulates mitophagy has been well-characterized in yeast and in mammalian cells and will not be discussed here since it has been extensively reviewed elsewhere [76,77]. During aging, mitochondrial dynamics have been reported to be affected in several tissues and organisms. For example, in ovarian germline stem cells (GSCs) from *D. melanogaster*, mitochondrial dynamics have been shown to shift toward fission, contributing to age-related GSC loss [78]. On the other hand, data on protein levels involved in mitochondrial dynamics during aging in skeletal muscle are quite discordant, with studies reporting a decrease [79,80,81], no change [82,83], and even an increase [84]. As aforementioned, fission is needed to isolate components of the mitochondrial network that need to be removed by mitophagy, a process that consists of the formation of double membrane vesicles around mitochondria in order to deliver the entire organelle to lysosomes for degradation [85]. Although the complete mechanism underlying the recruitment of the autophagosome to mitochondria has not yet been completely understood, decreased mitochondrial membrane potential has been shown to be one of the triggers [86]. Initial studies in *D. melanogaster* and HeLa cells have implicated PTEN-induced kinase-1 (PINK-1) and E3 ubiquitin ligase Parkin in mitochondrial removal via mitophagy [86,87,88,89]. Briefly, a decrease in mitochondrial membrane potential induces the stabilization and accumulation of PINK-1 on the OMM where it recruits and activates Parkin, which mediates the ubiquitylation of several mitochondrial proteins including mitofusin 1 and 2. The accumulation of poli-ubiquitin chains on the OMM are sensed by autophagy receptor proteins (e.g., optineurin (OPTN), neighbor of BRCA1 gene 1 (NBR1), TAX1 binding protein 1 (TAX1BP1), and p62), which bind to the ubiquitin chains and direct mitochondrial cargo to the autophagosome [90]. The autophagic flux has been shown to decrease during aging in several organisms and tissues, including the prematurely aging mtDNA mutator mouse [91], which in turn impairs the removal of damaged and impaired mitochondria, leading to mitochondrial dysfunction [92]. In this context, the upregulation of the autophagic flux by overexpression of the Atg5 gene, a mediator of the autophagy process, was found to have anti-aging features and to increase the mean lifespan in mice [93]. 

Other molecular pathways implicated in mitochondrial quality control have been shown to decrease with aging and affect mitochondrial function. For example, mitochondria retain a complex network of proteases that regulate protein synthesis, folding, degradation, and apoptosis. Several of these proteases, such as the ATP-dependent Clp protease proteolytic subunit (CLPP) and the Lon protease homologue (LONP), have homologues in bacteria, reminiscent of their mitochondrial origins. With advancing age, the ability of these proteases to maintain a healthy mitochondrial proteome decreases resulting in mitochondrial dysfunction [93,94,95]. Finally, the Ubiquitin Proteasome System (UPS) has been found to play a central role in the maintenance of mitochondrial homeostasis via regulation of fission/fusion, the proteome, and mitophagy, and with aging may become significantly deregulated [96]. 

**Figure 4:**
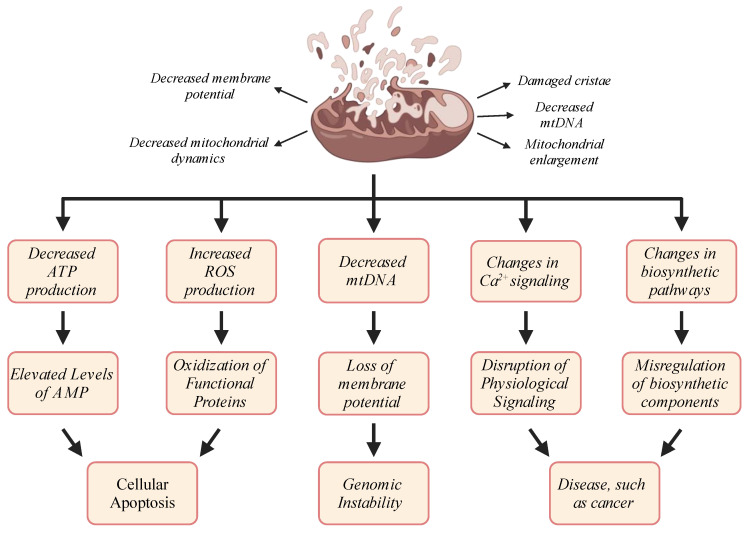
Biological cascades of mitochondrial dysfunction. Characteristics of mitochondrial dysfunction induce secondary attributes, such as decreased ATP production, increased ROS production, decreased mtDNA levels, changes in Ca^2+^ signaling, and changes in biosynthetic pathways, leading to tertiary attributes that result in cellular apoptosis, genomic instability, and disease. Created with BioRender.com accessed on 14 February 2024.

Taken together, the induction of mitochondrial dysfunction during aging appears to affect each of the pathways described herein and others, thereby multiplying the intensity of this dysfunction in a carom effect that accelerates quickly and explains the difficulty in finding effective therapeutic interventions to stop it once triggered (Figure 4).

## 4. Mitochondrial Dysfunction and Inflammation 

The mitochondrion has emerged as a key regulator of the innate immune system with mitochondrial morphology, metabolism, and physiology tightly controlling the immune cell fate during the innate and adaptive immune responses [97]. Furthermore, mitochondrial dysfunction has been linked to the onset of the chronic inflammation present in a plethora diseases, such as myocardial infarction (MI), rheumatoid arthritis (RA), and sickle cell disease, as well as neurodegenerative disorders [98,99]. Inflammation is a defense mechanism carried out by the immune system to fight microbial infections and tissue insults, as well as exogenous toxins. The inflammatory process follows a very well-defined program starting with the onset phase, which is elicited by an insult, followed by the acute and resolution phases, which aim to eliminate the danger and restore tissue homeostasis. When the inflammatory process, however, continues even after the insult has been neutralized or if the process occurs in the absence of an infection, commonly referred to as “sterile inflammation”, this may lead to chronic inflammation, a condition associated with aging and several age-related diseases, such as atherosclerosis and Alzheimer’s disease [100]. Chronic inflammation that is associated with aging has been termed “inflamm-aging”, and although its cause is unclear, it seems to be, at least in part, fueled by mitochondrial dysfunction [101,102]. 

Host cells can detect pathogenic molecules through the recognition of molecular motifs known as pathogen-associated molecular patterns (PAMPs) via pattern recognition receptors (PRRs), such as NOD-like receptors (NLRs) and toll-like receptors (TLRs) [103]. The activation of PRRs triggers several signaling pathways and results in the gene expression, synthesis, and release of a broad range of cell adhesion molecules, cytokines, and chemokines, which represent the first level of defense against infections and indicate an important link between the innate and adaptative immune responses [104]. Additionally, PRRs can recognize endogenous molecules when identified outside of their context, for example, when molecules are released into the extracellular space by stressed cells. These “self-molecules” are known as damage-associated molecular patterns (DAMPs) or alarmins. Similarly to PAMPs, DAMPs can elicit an innate immune response and induce inflammation [105]. Classical DAMPs include cytoplasmic DNA, heat-shock proteins, and mitochondrial components [105,106,107]. Specifically, mitochondrial components, due to their similarity to prokaryotic molecules, have been shown to act as very powerful alarmins when released from mitochondria into the cytoplasm [108]. Transcription factor A, mitochondrial (TFAM), mtDNA, mitochondrial ROS (mtROS), ATP, succinate, cardiolipin, as well as cytochrome *c* have all been described to act as mitochondrial DAMPs (mDAMPs) and can induce an inflammatory response by activating the innate immune system when present in the cytoplasm or extracellular space [104]. Mitochondrial DNA is one of the best studied mDAMPs, and numerous PRRs have been found to be able to bind to mtDNA within the cytoplasm, triggering a type I interferon (IFN-I) response [109,110]. For example, toll-like receptor TLR9 can bind to mtDNA and activate multiple signaling pathways, including pro-inflammatory nuclear factor kappa B (NF-κB) and the leucine-rich repeat (NLR) prying domain containing 3 (NLRP3) inflammasome, which induce an IFN-I response in several cell types, including macrophages, dendritic cells, and natural killer cells [111]. Furthermore, mtDNA activation of the NLRP3 inflammasome results in downstream activation of caspase-1 and subsequent cleavage and the activation of interleukin (IL)-1β and -18 [112,113]. The cyclic GMP-AMP synthase (cGAS), which recognizes aberrant dsDNA in the cytoplasm and mediates the synthesis of the second messenger cyclic dinucleotide 2′3′-Cyclic GMP-AMP (cGAMP), has also been shown to recognize and bind to mtDNA [114]. When bound to mtDNA, cGAS triggers a dimerization of STING proteins, inducing the phosphorylation of transcription factor interferon regulatory factor 3 (IRF-3) and interferon type I immune response with an expression of pro-inflammatory cytokines [115]. In this regard, it was shown that cytosolic release of mtDNA due to TFAM deficiency induced an IFN type I response via the cGAS/STING pathway in a TFAM heterozygous knockout mouse model [115]. The activation of the same pathway has also been reported in the mtDNA mutator mouse, which might contribute to its accelerated aging phenotype [116]. 

In addition to mtDNA, extracellular TFAM alone, or in the presence of other DAMPs, can synergistically increase the secretion of proinflammatory cytokines from immune cells [117,118]. ROS, which are normally produced during OXPHOS and have been shown to be elevated during mitochondrial dysfunction, have also been reported to activate the innate immune system [119]. High levels of mtROS have been shown to induce NLRP3 and promote the production of proinflammatory cytokines [120], and the inhibition of mtROS has been shown to block inflammasome-dependent IL-1β expression in macrophages [121]. ATP has also been found to play a key role in the inflammatory response by binding to purinergic (P2) receptors and alerting the immune system of tissue damage, which induces chemotaxis and phagocytosis [122]. P2 receptors are grouped into G-protein-coupled receptors, P2YR, and ligand-gated ion channels, P2XR, with the receptor subtype P2X-7 appearing to play a key role in various inflammatory diseases due to its ability to activate the NLRP3 inflammasome [123]. Succinate is a metabolic intermediate generated during the TCA cycle and has been shown to exert extra-metabolic functions, such as regulating gene expression and intracellular communication [124]. Succinate can also act as an mDAMP by inducing inflammation via the induction of IL-1β and has been found to directly interact and recruit dendritic cells by binding to the G protein-coupled receptor GPR91 [125,126]. Cardiolipin, a diphosphatidylglycerol lipid found exclusively in the mitochondrial inner membrane (IMM) and crucial for mitochondrial respiration and biogenesis, has also been shown to regulate inflammation and cell death [127,128]. Dead signals, infection, or cellular stressors have been shown to induce the translocation of cardiolipin to the cytosolic side of the outer mitochondrial membrane (OMM), thereby affecting mitochondrial function and the inflammatory response. In particular, oxidized cardiolipin (oxCL), but not native cardiolipin, was found to promote inflammation by increasing intracellular calcium concentrations in monocytes and macrophages resulting in leucotriene B4 synthesis via 5-lypoxygenase (5-Lox) [129]. Mitochondrial proteins, similar to bacteria, are N-formylated on their N-terminal methionine by the methionyl-tRNA formyltransferase (MTFMT) enzyme [130]. Mitochondrial formylated peptides (mtF-peptides) have been shown to evoke a pro-inflammatory response when recognized by formyl-peptide receptors (FPRs), a family of G-coupled transmembrane receptors comprising three subtypes, FPR-1-3 [131]. FPRs are expressed in various immune cells, such as neutrophils, macrophages, and natural killer cells, as well as dendritic cells, and are involved in regulating inflammation, chemiotaxis, and cell proliferation [131]. mtF-peptides are released by mitochondria due to tissue insults or pathological conditions, such as pulmonary, cardiovascular, and neurological diseases, and have been shown to have a negative effect on recovery by fueling a strong inflammatory response [132,133,134]. Lastly, cytochrome *c*, a nuclear-encoded electron carrier located in the IMM, has also been shown to have pro-inflammatory roles in neutrophils and monocytes by NF-κB activation [135]. 

Considering the numerous mitochondrial alarmins and their pro-inflammatory effects, it is important for cells to be able to sequester and isolate dysfunctional mitochondria before their contents are released into the cytoplasm or extracellular space, thereby eliciting an inflammatory response. Sequestration is mainly achieved by enclosing defective mitochondria in a double-membraned autophagosome via mitophagy for delivery to the lysosome for degradation. Indeed, it has been shown that when mitophagy becomes defective and impaired mitochondria are no longer isolated and removed, this results in inflammation and contributes to the etiology of several inflammatory and autoimmune diseases [136,137]. 

## 5. Mitochondrial Dysfunction and Brain Aging

During aging, the human brain undergoes morphological and functional changes that result in a loss of tissue mass as well as in cognitive decline [138]. Reduction in neuronal number due to cell death was once thought to be the main cause of the cognitive decline associated with aging; however, it is now evident that all cell types in the brain are equally affected by aging and their dysfunction contributes to cognitive decline. For example, the recruitment of oligodendrocytes has been shown to decrease during aging, leading to a loss in myelination [139]. Additionally, gene expression deregulation in astrocytes has been shown to affect synapse elimination and glutamate uptake during aging [140]. Furthermore, microglia have been shown to become activated during aging, thereby contributing to brain inflammation [141]. Interestingly, it seems that morphological changes at the neuronal level, such as a decrease in dendritic spines, alterations in axonal morphology, and accumulation of intracellular granules made of protein aggregates and lipofuscin, have a more prominent role in age-related cognitive decline than neuronal loss in humans [142]. For practical reasons, it is complicated to procure aged human tissues, let alone brain tissue samples, to study the changes that occur at the cellular level, thus most studies use postmortem tissues, which present several limitations including sample availability and tissue preservation [143]. To overcome these limitations, rodents have been widely used to model human brain aging in order to study the age-associated molecular and cellular processes [144]. 

Despite the human brain weighing only 2% of the total body weight, almost 20% of the basal oxygen is consumed by this organ in order to produce enough energy for the approximately 86 billion neurons and 85 billion glial cells that comprise it [145]. Glucose is the main source of energy in the adult brain and its oxidation produces ATP almost entirely through OXPHOS in the mitochondria, thus underpinning the importance of this organelle for brain homeostasis [146]. Energy is constantly required to sustain the synthesis of neurotransmitters as well as to maintain the membrane potential needed for action potential propagation and synaptic transmission, including the re-uptake of neurotransmitters from the synaptic cleft [147].

A large body of evidence demonstrates that bioenergetic impairments as well as disturbances in the OXPHOS machinery occur in the brain during aging [148]. Although efficient, OXPHOS produces ROS as a byproduct, and the brain is especially susceptible to oxidative damage because it contains a plethora of oxidizable substrates, such as fatty acids, an abundance of catalytic transition metals, and a high rate of oxygen consumption per gram of tissue [149]. Several studies have demonstrated an association between the oxidative damage of DNA (8-OH-dG), lipids (MDA and 4-HNE), and proteins (carbonyls and protein 3-nitrotyrosine) with brain aging [150,151,152]. Notably, when markers of oxidative damage were measured in the aged brain, distinct regions showed different susceptibility to oxidative damage with midbrain, striatum, and hippocampus having higher levels of protein carbonylation as compared to cortex and cerebellum [153]. Similarly, brain region-specific alterations have also been reported in response to mitochondrial dysfunction, with the cortex being better protected than the striatum [154]. Moreover, mitochondria located in the synaptic nerve terminals have been shown to be more sensitive to oxidative damage and age-related mitochondrial dysfunction, as compared to non-synaptic mitochondria [155]. In this regard, an abundance of oxidatively-modified mitochondrial proteins related to energy metabolism, such as ATP synthase and pyruvate kinase, have also been found in cortex and hippocampus from aged rats [156]. It has been proposed that the impairment of brain mitochondrial function during aging might be the result of the decreased electron transfer rate of Complex I [157] and IV [158]. Interestingly, gene expression of mitochondrial subunits for Complexes I, III, IV, and V have been found to be down-regulated in old *TG2576* mice [159] and *Ndufs4* knock-out mice [160], models of Alzheimer’s disease pathology and of Complex I deficiency, respectively.

Importantly, the effects of neuronal oxidative stress are normally counteracted by a well-developed antioxidant system; however, during aging the antioxidant defense system may become overwhelmed [161]. A shift to a pro-oxidized state, determined by a decrease in the GSH/GSSG ratio, with GSH serving as the body’s “master” antioxidant and GSSG as the oxidized form of GSH, was found in forebrain and cerebellum from 21 month-old mice, as compared to 3 month-old controls [162]. As the brain ages, the effects of oxidative stress on mtDNA may lead to mutations and deletions and subsequently impair the OXPHOS complexes, increase ROS production, and further exasperate oxidative stress levels [163,164]. This vicious cycle may lead to decreased energy supply, increased susceptibility to apoptosis, and a progressive decline in tissue function [165]. A 10-fold increase in mtDNA levels of 8-OHdG as well as elevated mtDNA point mutations and deletions in frontal cortex, substantia nigra, and putamen from elderly individuals above the age of 67 have been reported [68,166,167]. 

Mitochondrial quality control mechanisms, such as fusion, fission, and mitophagy, are important processes used to preserve cells against damage [168]; however, reports indicate that as the mtDNA mutation load increases during aging these processes may begin to lose their efficiency. For example, Drp1, a protein essential for mitochondrial fission, has been shown to be down-regulated in old C57BL/6 mice [169], and its removal in adult mouse forebrain resulted in altered mitochondrial morphology and mitochondrial transport to the synapse, as well as decreased oxygen consumption and ATP production [170]. Importantly, these findings suggest that mitochondrial dynamics, mitophagy, and biogenesis become impaired during aging, and may be involved in the pathogenesis of various neurodegenerative diseases. 

Mitochondrial dysfunction also contributes to calcium buffering dysregulation, which is associated with brain aging and affects neuronal synapses, thus, paving the way for the onset of neurodegenerative diseases [171]. Taken together, these studies emphasize that brain aging is marked by decreased OXPHOS, increased mtDNA mutation load, increased oxidative stress levels, saturation of antioxidant defenses, dysregulation of calcium homeostasis, and impaired mitochondrial quality control mechanisms, which may create the milieu for the onset of neurodegenerative disorders.

## 6. Role of Mitochondrial Dysfunction in Brain Aging Disorders

In broad terms, brain aging disorders are defined as a heterogeneous group of diseases characterized by the progressive and selective deterioration of anatomically and physiologically related neuronal systems [172]. Although the underlining cause for most neurological disorders remains unclear, mitochondrial dysfunction, oxidative stress, and the accumulation of oxidative damage have been strongly implicated in several brain aging disorders (see Table 1); however, a clear understanding of the relationship between mitochondrial dysfunction, mtDNA mutations, and the aging phenotypes seen within these brain aging disorders is only beginning to emerge [173]. Here, we will discuss only a few disorders, focusing on Huntington’s disease (HD), Parkinson’s disease (PD), and Alzheimer’s disease (AD), examining what has been learned over the years regarding the role of mitochondrial dysfunction in disease onset and the progression of those diseases using animal models.

## 7. Huntington’s Disease

Huntington’s disease (HD) is an autosomal dominant hereditary neurological disorder caused by the expansion of trinucleotide CAG repeats in the gene coding for the protein huntingtin (HTT), which translates as a polyglutamine repeat in the HTT protein [174]. Patients with HD have between 36 to more than 120 CAG repeats, with the repeat length correlating to symptom severity and the age of disease onset [188]. HD is characterized by involuntary postures and abnormal movements (chorea, dyskinesia, and dystonia) of the legs, face, and trunk, as well as speech disturbances (dysarthria) and psychiatric abnormalities (mood changes, psychiatric disturbances, and cognitive deficits). As disease progression continues, motor rigidity and dementia ultimately predominate, leading to death approximately 15–20 years after disease onset [189]. Mutant HTT selectively affects medium-sized spiny striatal projection neurons and results in the depletion of their neurochemical components, with cerebral cortex affected later within disease progression [175,190]. In patients with HD, selective medium spiny neuronal loss has been detected in brain regions, including caudate nucleus and putamen within striatum, pyramidal neurons of cerebral cortex, and, to a lesser extent, neurons within subthalamus and hippocampus [176]. Although the molecular mechanism of mutant HTT-induced toxicity has not been completely understood, it has been postulated that the mutant protein might trigger a cascade of both compensatory and defective genetic programs and molecular processes that promote transcriptional damage and mitochondrial dysfunction, such as altered mtHtt-induced p53 transcriptional activity, deficits in mitochondrial trafficking, and abnormal fission and fusion processing [191]. In turn, this phenomenon leads to increasingly fragile neurons that are more vulnerable to endogenous stresses, such as oxidative damage, the expression of inflammatory signals, excitotoxic stress, pro-cellular apoptotic signaling, and energy depletion, all of which are hypothesized as playing a key role in the neuronal death seen within HD disease progression [177].

More than three decades ago, evidence of mitochondrial dysfunction in HD was first observed in ultrastructural studies on brain biopsies from HD patients [192]. Consistent with these observations, ETC complex subunits have been reported to be highly involved in the selective degradation of medium-sized spiny striatal neurons, suggesting that mutant HTT may alter nuclear-encoded components of the mitochondrial ETC and lead to primary mitochondrial dysfunction. Furthermore, considerable reductions have been reported in the functioning of mitochondrial Complexes II, III (severe), and IV (mild) in neostriatum from HD patients [189] (Table 1). Mutant HTT has also been suggested to induce mitochondrial dysfunction by decreasing mitochondrial Ca^2+^ loading capacity and leaving neurons more vulnerable to Ca^2+^-mediated toxicity [193]. Furthermore, it has been suspected that mitochondrial fusion and fission may become altered as HD progresses, resulting in mitochondrial dysfunction and, eventually, neuronal death [177]. A plethora of evidence points to mitochondrial bioenergetic deficits occurring in HD patients, such as: pronounced weight loss regardless of a sustainable caloric intake, increased levels of lactate in cerebral cortex and basal ganglia, decreased activity of mitochondrial Complexes II, III, and IV, a decreased number of mitochondria in postmortem brain, impaired mitochondrial respiration, reduced ATP production, reduced aconitase activity in basal ganglia, abnormal mitochondrial morphology in cortex, progressive atrophy and decreased glucose utilization in striatum, and abnormal mitochondrial membrane depolarization in lymphoblasts [176]. Taken together, it is evident that mitochondrial impairment plays a critical role in HD, thus therapeutic strategies targeting mitochondrial dysfunction may lessen disease progression and improve outcome.

## 8. Mitochondrial Dysfunction in Models of Huntington’s Disease

Alongside human postmortem brain samples, animal models have proven to be useful tools to investigate the mechanisms of mitochondrial dysfunction in HD. One of the first HD-focused models is the R6/2 transgenic knock-in (KI) mouse, which expresses a portion of the human HD gene under human gene promoter elements (1 kb of 5′ UTR sequence and exon 1 together with approximately 115–150 CAG repeats) [194]. These mice show subtle learning and motor deficits at 1 month post-natal, accumulating nuclear inclusions throughout the brain starting as early as 3 weeks of age, and die between 3 and 4 months of age [195]. Notably, the same type of inclusions have been observed in postmortem brains from HD patients, illustrating a comparison between the R6/2 model and human patients [195]. R6/1 mice, which contain fewer CAG repeats (approximately 120) and are complementary to R6/2 mice, present with similar symptomatology but have a milder phenotype and slower disease progression [196]. Unlike the R6/2 model, R6/1 mice also exhibit depression-like symptoms, demonstrated by the tail suspension and Porsolt forced swim paradigms [197]. Both R6/2 and R6/1 mice have demonstrated deficiencies in mitochondrial function, such as a significant reduction in Complex II and Complex IV flux control ratios and a significant increase in oxidative stress and fragmented mitochondria in striatum [198,199]. In addition to the R6 models, the yeast artificial chromosome (YAC) mouse was introduced to specifically model the effect that *HTT* CAG repeat length (46, 72, or 128 CAG repeats) has on disease progression [200]. As demonstrated in the human population, YAC mice with more CAG repeats exhibit more severe phenotypes, with YAC128 mice exhibiting atrophy in several brain areas, including striatum, hippocampus, and cerebellum, and developing marked cognitive deficits as well as hypoactivity [201]. In comparison, YAC46 and YAC72 mice exhibited similar symptomatology, albeit to a lesser extent and later in life [202]. Similar to R6 mice, YAC128 mice have been shown to illustrate mitochondrial function deficiencies, such as decreased mtDNA copy number and impaired mitochondrial-dependent Ca^2+^ handling [203,204]. Considering that R6/1, R6/2, and YAC mouse models all contain expanded CAG repeats in the *HTT* gene, researchers continue to use these mouse models as tools to study the relationship between the *HTT* mutation, mitochondrial dysfunction, and associated phenotypes to better understand the etiology of HD.

## 9. Parkinson’s Disease

Parkinson’s disease (PD) is the most common movement disorder and second most common brain aging disorder, only after Alzheimer’s disease [205]. PD is indicated primarily by the loss of dopaminergic neurons in substantia nigra pars compacta (SNpc), resulting in a dopamine deficiency in basal ganglia. PD is characterized by an accumulation of protein aggregates known as *Lewy bodies*, composed chiefly of α-synuclein, ubiquitin, neurofilaments, and molecular chaperones. PD symptomatology is prominently encompassed by motor deficits, including bradykinesia, hypokinesia, resting tremor, muscle rigidity, and postural instability, as well as by neurological deficits, including autonomic dysfunction, sleep disturbances, depression, and cognitive impairment [206]. There are two forms of PD: sporadic and familial, where familial forms have an expected family history and introduce a genetic component of the disease, while sporadic forms occur randomly and can arise as a result of an environmental or undetermined genetic stressor [207]. Interestingly, familial forms only account for approximately 10% of PD diagnoses and show an early onset of symptoms. Considering that the sporadic form of PD accounts for almost 90% of cases, the etiology of PD remains uncertain. As a result, there are several hypotheses that aim to explain this phenomenon. For example, the environmental hypothesis suggests that neurodegeneration inducing PD-like symptoms results from exposure to a dopaminergic neurotoxin. Indeed, the progressive neurodegeneration seen in sporadic forms of PD may result from chronic or limited exposure to neurotoxins, with each incident ultimately leading to PD-like symptomatology. Alternatively, an endogenous toxin may contribute to the onset of PD-like symptoms. Specifically, alterations in metabolism resulting from exposure to environmental toxins or from modifying genetic factors may result in the creation of toxic substances, such as ROS, and induce mitochondrial dysfunction [178].

In 1983, the first link between mitochondrial dysfunction and PD became apparent when MPTP (1-methyl-4-phenyl-1,2,3,6-tetrahydropyridine) was found to cause PD-like symptomology in intravenous drug users [208]. Once MPTP crosses the blood-brain barrier (BBB), it is transformed into its toxic form, 1-methyl-4-phenylpyridinium (MPP+), by monoamine oxidase (MAO). MPP+ specifically interferes with Complex I activity in substantia nigra dopaminergic neurons in both humans and animal models.

Since this original finding, several neurotoxins, such as rotenone insecticide and organochlorine pesticides, have been identified as potentially contributing to the sporadic form of PD, raising an awareness of the association between environmental exposure with risk of developing PD later in life [209]. Studies of both MPTP exposure-induced PD and familial PD have confirmed that mitochondrial impairment plays a key role in disease onset and progression. In postmortem studies, varying degrees of Complex I and II deficiency were found in individual substantia nigra neurons from PD patients, with approximately 60% deficiency in Complex I and approximately 65% deficiency in Complex II [210]. A widespread reduction in Complex I expression has also been observed in multiple brain regions from patients with PD, including hippocampus, pedunculopontine nucleus, and putamen [179].

To date, a handful of genes have been identified in causing familial PD, with mitochondrial dysfunction resulting from the mutation of many of these genes. One such gene, *PRKN*, was identified in 1998 and represents the most common cause of autosomal recessive PD with juvenile onset [211]. Parkin is a cytosolic E3 ubiquitin ligase that ubiquitinates target proteins and results in either proteasomal degradation or activation of signaling pathways. Residing in the cytosol, Parkin mostly functions alongside the mitochondria [212]. One of the first links between *PRKN* gene mutation and mitochondrial dysfunction was discovered when Darios and colleagues observed that an overexpression of Parkin prevents cytochrome *c* release and results in mitochondrial swelling in ceramide-treated cells. Furthermore, it was also found that a small fraction of Parkin is able to physically interact with the OMM. Although its mechanism is still under investigation, it is suspected that Parkin may serve to protect cells from mitochondrial toxins and become impaired [213], thus, mutation of the *PRKN* gene is predicted to play a key role in the mitochondrial dysfunction reported in familial forms of PD.

*PINK1* is another gene linked to familial PD and mutation of the *PINK1* gene is found to be the second most common mutation in causing autosomal recessive forms of PD. The relationship between PINK1 deficiency and mitochondrial dysfunction is multifaceted and includes events such as a decrease in mitochondrial membrane potential, the development of abnormal mitochondrial morphology, a reduction in Complex I and IV activity, a decline in mitochondrial transport and mtDNA levels, a decrease in ATP production, and an increase in ROS production [33]. Furthermore, the disruption of Complex I activity is an early event in PINK1 deficiency, ultimately causing the organism to develop an increased sensitivity to apoptotic stress, an influx in mitochondrial membrane depolarization, and a deficit in synaptic transmission [180]. Interestingly, shortly after PINK1 deficiency was linked to families with recessive PD, it was reported that *PINK1* KO and *PRKN* KO in *D. melanogaster* have similar phenotypes, thus suggesting that these two proteins function using the same genetic pathway [214]. As discussed above, molecular studies have revealed that PINK1 accumulates on impaired mitochondria, indicating that PINK1 works as a sensor for mitochondrial dysfunction, and recruits Parkin from the cytosol to label damaged mitochondria for degradation via mitophagy [215]. 

*DJ-1* mutations are thought to contribute to autosomal recessive forms of PD and interrupt proper mitochondrial functioning. DJ-1 can translocate to mitochondria from the cytosol in response to mitochondrial stress, suggesting a role in regulating protein trafficking and mitochondrial metabolism. It has been found that mutation of the *DJ-1* gene alters mitochondrial morphology and function [181]. Furthermore, animals carrying a mutation in the *DJ-1* gene showed increased sensitivity to neuronal toxins [216], and DJ-1 has been found to be crucial for normal lifespan, motor functioning, and neuronal resistance to oxidative stress and damage in several organisms [209].

*ATP13A2* encodes a lysosomal type 5 P-type ATPase and is thought to contribute to autosomal recessive PD. The ATP13A2 protein has been identified in several cellular vesicles, such as lysosomes and autophagosomes, and has been proposed to participate in the autophagy-lysosomal pathway (ALP). Mutations in the *ATP13A2* gene have been associated with autosomal recessive levodopa-responsive early-onset parkinsonism, or Kufor–Rakeb syndrome (KRS). Patients receiving a diagnosis of KRS typically present with PD symptomology alongside other manifestations, including supranuclear gaze palsy, spasticity, and facial-faucial myoclonus [217].

*SNCA* encodes α-synuclein (α-syn), a small 140 amino acid polypeptide, and mutation of the *SNCA* gene has been linked to the autosomal dominant form of PD. Although its function is still largely unknown, α-syn is thought to mediate neurotransmitter release at presynaptic terminals and interact with membranes of several organelles, including mitochondria. Additionally, α-syn has been found to have a non-canonical mitochondrial targeting sequence and has been shown to influence mitochondrial structure and function. α-syn is thought to contribute to mitochondrial dysfunction in PD by inhibiting Complex I activity and increasing ROS production [218]. 

*LRRK2* gene mutations are also thought to contribute to the autosomal dominant form of PD. LRRK2 co-localizes with several regulators of mitochondrial fusion and fission in the cytosol or on the mitochondrial membrane [218]. The inhibition of LRRK2 is thought to contribute to mitochondrial dysfunction by increasing oxidative stress, decreasing ATP production, decreasing mitochondrial membrane potential, causing the elongation and fragmentation of mitochondria, increasing mitophagy, and increasing mtDNA damage [219].

Currently, no mtDNA mutations have been found to cause PD, despite that several subunits that comprise the ETC are encoded by mtDNA. On the other hand, mitochondrial dysfunction in PD is thought to potentially arise from mutation of the mtDNA maintenance gene, *POLG*, which encodes the mtDNA polymerase and helicase [220,221]. Studies crossing Parkin or *DJ-1* KO mice with the mtDNA mutator mouse did not induce a Parkinsonian phenotype [222,223]; however, further evaluation is still warranted. Considering that age is the greatest risk factor for developing PD and that the aging process is involved with a reduction in mitochondrial functioning, mtDNA mutations and respiratory-based mitochondrial impairment may play a key role in PD pathogenesis by increasing the vulnerability of the organism to other environmental and genetic stresses [207].

## 10. Mitochondrial Dysfunction in Models of Parkinson’s Disease

The essential requirement for disease-based, etiological models of PD is for adult onset of relatively progressive and specific dopaminergic neuronal loss. Additionally, behaviors that involve striatal functioning, such as the ability to learn a stimulus-response paradigm or the habituation to a novel environment, may be useful in evaluating striatal dopaminergic neuronal functioning. Pathological models do not depend on disease-related genes and use toxins or non-PD related genetic mutations to imitate the selective degeneration of dopaminergic neurons in substantia nigra. To this end, a plethora of neurotoxins, including 6-hydroxydopamine (6-OHDA), MPTP, paraquat, and rotenone, have been used to induce PD-like symptomatology in both vertebrate (zebrafish, mouse, rat, monkey) and invertebrate (*D. melanogaster*, *C. elegans*, snails) organisms. These neurotoxins have received the most attention for their ability to provoke high levels of ROS within the brain, along with nigrostriatal dopaminergic neurodegeneration, Complex I inhibition, and PD-like symptomatology [224,225]. Among human postmortem tissue, animal models have also been useful in identifying the mechanisms underlying mitochondrial impairment within PD. 

Knock-out (KO) mouse models of PRKN, PINK1, and DJ-1 have been generated to evaluate autosomal recessive forms of PD. Surprisingly, despite both *PRKN* and *PINK1* being recessively inherited and *PRKN* mutations accounting for about 50% of familial PD cases, PRKN and PINK1 KO mice do not demonstrate any abnormalities in dopaminergic neurons or dopamine levels, nor do they exhibit dysfunction in locomotion [226]. However, both PRKN and PINK1 KO mice display deficits in nigrostriatal dopamine transmission and demonstrate mild mitochondrial abnormalities [227]. Likewise, DJ-1 KO mice have not been found to show any behavioral abnormalities, with dopamine levels and dopaminergic neuronal functions remaining normal. Nonetheless, neurotransmission in the nigrostriatal circuit and mitochondrial abnormalities of DJ-1 KO mice appear to be similar to PRKN and PINK1 KO mice [228]. However, with no loss of dopaminergic neuronal function, the PRKN, PINK1, and DJ-1 KO mouse models have little utility outside of laboratory research and may not be useful in testing possible therapeutic strategies for PD [229]. Alongside KO mouse models of *PRKN*, *PINK1*, and *DJ-1*, models of *SNCA* and *LRRK2* gene mutations have failed to exhibit the basic phenotypes seen with PD, such as resting tremor, bradykinesia, rigidity, and postural instability; thus, their utility lies in identifying genetic and pharmacological modifiers of α-synuclein-induced neurodegeneration [227].

The MitoPark mouse is a transgenic model utilized in studying PD and includes the selective removal of the mitochondrial transcription factor, TFAM, from midbrain dopaminergic neurons, resulting in severe mitochondrial respiratory defects and ultimately cell death [230]. Notably, this model recapitulates the selective and progressive loss of dopaminergic neurons in substantia nigra and ventral tegmental area, along with decreased monoamide levels in striatum, the growth of inclusion bodies, the development of cognitive impairment leading to motor deficits, and the establishment of spontaneous horizontal and vertical locomotor deficits [231]. At approximately 14 to 15 weeks of age, MitoPark mice demonstrate decreased locomotion and exploratory behavior, leading to limb rigidity at 20 weeks of age, with it being necessary to euthanize mice at approximately 45 weeks of age due to poor health [230]. When evaluated in the open-field behavior test, MitoPark mice displayed decreased horizontal and vertical (rearing) behaviors as early as 24 weeks of age. These mice also exhibited decreased levels of dopamine and its metabolites, 3,4-Dihydroxyphenylacetic acid (DOPAC) and homovanillic acid (HVA), in striatum and anterior cortex, correlating with behavioral data and mimicking the symptomatology observed in human PD patients [232]. Although the MitoPark mouse has been found to be the most useful transgenic model in evaluating PD progression as well as response to therapies such as L-DOPA [232], the model’s translatability is still questionable, considering that alterations in *TFAM* have not been confirmed in human PD pathophysiology. 

## 11. Alzheimer’s Disease

Alzheimer’s disease (AD) is currently the most common cause and leading form of dementia world-wide, affecting over 25 million people today and expected to reach over 100 million by 2050 [182]. AD is currently the most common brain aging disorder, indicated by the progressive age-dependent loss of memory, increased prevalence of extracellular amyloid plaques that originate from the processing of amyloid precursor protein (APP), and intracellular neurofibrillary tangles (NFTs) made from hyperphosphorylated tau protein (pTau) in the CNS. These plaques contain amyloid beta (Aβ) fibrils that remain as prognostic indicators of AD [172] and it is not apparent as to whether Aβ and pTau drive the pathophysiology of AD or are the result of other common underlying processes [183]. Similar to PD, AD comes in two forms: familial and sporadic, with approximately 1% of cases associated with familial autosomal dominant mutations in the genes encoding for amyloid beta protein precursor (*AβPP*) or presenilin 1 or 2 (*PS1* or *PS2*), which are directly involved in AβPP processing [184]. Most AD cases are found to be sporadic, leaving the origin of the disease to remain in question, but it is suspected to result from the interaction of a multitude of factors, including environment, lifestyle, genetics, and age. Despite the common occurrence of the sporadic form, most AD research focuses on the familial form since its etiology is the most straightforward to model [183]. Mitochondrial dysfunction has been hypothesized as being one of the leading causes of cognitive abnormalities seen within AD patients and aging phenotypes; however, the connection between mitochondrial impairment and Aβ plaque formation in AD pathology is still in question [185].

In the 1980s, the deficiency of two Krebs cycle enzymes, pyruvate dehydrogenase complex (PDC) and ketoglutarate dehydrogenase complex (KDC), was discovered in brains from AD patients, indicating that mitochondrial function may be affected within Alzheimer’s disease progression. Less than a decade later, a reduction in cytochrome oxidase (Complex IV) activity was reported in platelet mitochondria from an AD patient, and was later confirmed also in the AD brain [186]. Additional evidence revealed that mitochondrial dysfunction is an early and noticeable feature of AD, with lower energy metabolism serving as one of the best-known primary abnormalities of the disease [233,234]. Specifically, several key enzymes of mitochondrial metabolism, including KDC, PDC, and Complex IV, have been found to have decreased expression and activity in AD [235]. Furthermore, profound mitochondrial abnormalities, such as changes in mitochondrial number, enzyme activity, and morphology, have been reported in brain from patients with sporadic AD [236]. Cumulating evidence illustrates that the regulation of mitochondrial function and turnover becomes defective and contributes to AD pathophysiology, resulting in reduced mitochondrial membrane potential, increased mitochondrial permeability, and increased ROS production, thus leading to the damage of proteins, lipids, nucleic acids, and, ultimately, cell death [187]. Additionally, alterations of mitochondrial cristae and intra-mitochondrial accumulations of osmiophilic material are prevalent. There is also an increased range of mitochondrial sizes in AD, with higher numbers of smaller mitochondria alongside an increase in enlarged mitochondria.

The pathophysiology of AD is mainly characterized by the deposition of Aβ plaques and NFTs within the brain. Aβ plaques have been found to deplete Ca^2+^ stored in endoplasmic reticulum (ER), resulting in an accumulation of Ca^2+^ in the cytosol. In response to this accumulation, endogenous glutathione (GHS) levels become reduced, leaving the cell with an abundance of ROS. ROS-induced oxidative stress is beginning to emerge as a prominent factor in AD pathogenesis, with ROS overproduction predicted to play a key role in the accumulation and deposition of Aβ plaques [237]. Additionally, it has been found that oxidative stress occurs early within Alzheimer’s disease progression, even proceeding Aβ plaque deposition. Oxidative stress can induce an accumulation of Aβ plaques in vivo and in vitro. Adversely, Aβ plaques have been found to impair mitochondrial respiration and induce oxidative stress [238]. Due to the discovery of both APP and Aβ in the mitochondrial membranes, growing evidence suggests that elevated Aβ levels may contribute to the mitochondrial impairment seen in AD. Moreover, Aβ and APP have been found to interact with mitochondrial proteins and may affect mitochondrial fusion and fission forces [239], impair mitochondrial transport, disrupt the mitochondrial ETC [240], and impair overall mitochondrial function [241]. Unfortunately, the true relationship between Aβ plaques and mitochondrial dysfunction is still unknown, leaving the question as to whether mitochondrial dysfunction may cause an accumulation of Aβ plaques or vice versa, or if both play a causative role for each other.

## 12. Mitochondrial Dysfunction in Models of Alzheimer’s Disease

A large amount of insight about the characteristics of AD pathophysiology has been gained by using animal models [242]. Although a small fraction of AD diagnoses result from the autosomal dominant form, its pathology and symptoms have pronounced similarities to that of the sporadic form, thus mutations of the three genes, *APP*, *PS1*, and *PS2*, have been used as the basis for most AD mouse models [243]. Furthermore, researchers have been able to generate mouse models with characteristics of the sporadic form, such as the APOE4 mouse, and have exposed these transgenic mouse models to conditions that are associated with sporadic AD, such as a high-fat diet, obesity, and hypertension [244,245].

The *APP/PS1* transgenic mouse has been generated to model the autosomal dominant early onset form of AD and is often used to analyze the neurochemical changes associated with AD [246]. This model contains mutations in the human amyloid precursor protein (*APP*) and presenilin 1 (*PS1*) genes, resulting in an increased production of the amyloidogenic form of Aβ peptide at 6 months of age, followed by profound memory loss, as well as cognitive and behavior changes shortly thereafter [247]. The APP/PS1 mouse model has been shown to illustrate deficiencies in mitochondrial function, such as an increase in mitochondrial oxygen consumption, a decrease in ATP production, and high levels of ROS [248,249].

In addition to the *APP/PS1* mouse, the Tau/Harlequin double mutant mouse serves as a model to predominantly study mitochondrial dysfunction and human AD-associated tau pathology, as it includes the genetic makeup of the Harlequin mouse, the neurodegenerative aspects of Complex I deficiency, and the tau pathology commonly seen in AD [197]. This model is generated by crossing the Harlequin, or *Aifm1^Hq^* mouse, which has been established as a model of neurodegenerative-associated mitochondrial dysfunction and oxidative stress [250], with a mouse expressing the human mutant *tauP301L* gene that results in widespread human-like tau filament formation in the brain [251]. Importantly, the combination of these two mutant genes results in increased tau pathology, increased neurodegeneration, decreased OXPHOS activity, and decreased ATP production [250]. 

To evaluate the effects of both Aβ and pTau on AD progression, the triple mutant 3xTG-AD mouse expresses the mutant genes *APPswe*, *PS1M146V*, and *tauP301L*, and exhibits both extracellular Aβ plaques and intracellular neurofibrillary tangles as well as severe cognitive deficits [252]. These mice also present with decreased vitamin E and glutathione levels, leaving these mice more susceptible to oxidative stress due to their decreased level of endogenous antioxidants [253]. Consequently, 3xTG-AD mice have been shown to have high levels of oxidative stress, decreased mitochondrial respiration and PDC levels, and changes in mitochondrial dynamics [254]. 

Considering that the autosomal dominant forms of AD are quite rare, researchers have aimed to study the common sporadic form of AD. Sporadic AD risk factors are modulated by several genes, with the strongest risk factor gene being the *APOE* gene, which encodes apolipoprotein E (ApoE), a protein responsible primarily for lipid storage and metabolism [255]. There are three alleles of *APOE*, where ε2 is protective, ε3 is the most common and has no effect, and ε4 is associated with an increased risk of developing AD [256]. The ApoE4 allele has both Aβ-dependent and Aβ-independent roles within AD, with both roles serving to give important insight into the true nature of AD pathogenesis [243]. To model the sporadic form of AD, the humanized APOE4 mouse was created, which contains a homozygous knock-in human *APOE4* allele pair and expresses the human apolipoprotein E4 isoform under the control of the murine Apoe regulatory sequences. Importantly, this mouse expresses the phenotype of late-onset AD, exhibiting Aβ and pTau accumulation, progressive memory loss, and cognitive decline, closely modeling the AD-like phenotype observed in humans [257]. The APOE4 model presents with a variety of mitochondrial dysfunction, such as reduced fission and mitophagy dynamics, decreased mitochondrial respiratory capacity, and reduced expression of all mitochondrial ETC complexes [258].

## 13. Current Treatments of Mitochondrial Dysfunction 

Given the importance of mitochondrial dysfunction in the pathogenesis of brain aging disorders, therapeutics targeting mitochondrial dysfunction have been investigated as possible treatments. Although the pathological phenotypes associated with endogenous and exogenous risk factor models may be different, the existence of mitochondrial dysfunction, alongside mitochondrial morphology impairment, increased ROS levels, and the deficiency of the mitochondrial ETC, all seem to be common pathways. Some treatments aimed at targeting mitochondrial dysfunction may affect all brain aging disorders, while some therapies may only be used in the treatment of a specific disease. Here, we provide a brief overview of various pharmacological and lifestyle therapeutics that are used today and are aimed at targeting mitochondrial dysfunction in aging and brain aging disorders. 

## 14. PGC-1α Expression 

Interestingly, PGC-1α is increasingly being recognized as an important target for brain aging disorders. PGC-1α is a transcriptional coactivator that regulates the genes involved in cellular metabolism and remains as the central inducer of mitochondrial biogenesis [259]. PGC-1α expression and function have been found to be defective in all brain aging disorders; therefore, the pharmacological and transcriptional activation of the PGC-1α pathway is expected to have neuroprotective qualities. Interestingly, an overexpression of PGC-1α was shown to reduce Aβ plaque formation in an in vitro model of AD as well as stabilize the mitochondrial membrane and reduce mitochondrial toxicity in an in vitro model of HD [260]. Further, when overexpressing PGC-1α in skeletal and cardiac muscle in the mtDNA mutator mouse, an increase in mitochondrial biogenesis and function occurred, improving heart and muscle function despite not improving the increased mtDNA mutation burden [73]. Thus, the expression of PGC-1α offers the potential to preclude mitochondrial dysfunction in aging and age-related disorders. 

## 15. Caloric Restriction and Fasting Diets

Currently, caloric restriction (CR) is the most effective non-genetic intervention that has been found to extend lifespan across several species and reduces the risk of developing a number of diseases in both primates and non-primates, including diabetes, cancers, sarcopenia, cardiovascular diseases, and hearing loss [261]. Moreover, experimental data have shown that CR decreases the age-associated accumulation of oxidative damage, reduces the steady-state level of oxidative stress, and increases metabolic potential [262]. Additionally, CR is thought to slow the rate of aging and reduce the risk of many age-related diseases, including brain aging disorders, via the protection of many mitochondrial components, including the mitochondrial genome, i.e., mtDNA [263].

Physiologically, CR alters the way in which available energy sources in food are processed by the body. For example, in caloric excess, molecules such as carbohydrates are used for energy resulting in fat storage, primarily because they have a higher energy content per gram. Nevertheless, during CR, all energy sources are used, including fats and amino acids, and the organism minimizes carbohydrate use in peripheral tissue to maintain adequate blood glucose levels for the brain. Furthermore, during CR, energy-producing tissues, e.g., muscle, shift towards the β-oxidation of fatty acids to obtain energy, a process that occurs entirely in mitochondria [264]. 

To further investigate the ability of CR to improve the human lifespan, several studies have been conducted, specifically in non-human primates, such as the rhesus monkeys [265]. Despite the findings being contradictory, most likely due to differing control diets, study design, and study implementation, a direct comparison of longitudinal data from two landmark studies [266,267] confirms that the health benefits of CR are conserved in monkeys, suggesting that CR mechanisms are likely to be beneficial in humans [265]. Recent studies in humans indicate that CR started at any point in life, even in adulthood, may reduce the risk of age-related disorders, enhance healthy aging, and slow the overall pace of aging [268,269]. Based on the current literature, it is now suggested that daily caloric intake should be decreased by at least 20 to 40 percent in order to successfully reduce aging phenotypes and increase longevity. 

Alongside caloric restriction, intermittent fasting, including alternate day fasting, periodic fasting, time-restricted feeding, and fasting-mimicking diet, is being evaluated for its efficacy in targeting mitochondrial dysfunction and prolonging lifespan. Findings from animal studies show that intermittent fasting is able to not only increase both mean and maximum lifespan [270], but is also beneficial to models of Alzheimer’s and Parkinson’s disease, ischemic stroke, autism spectrum disorder, and mood and anxiety disorders [271]. Additional evidence indicates that intermittent fasting enhances long-term memory consolidation and hippocampal neurogenesis [272] as well as reverses age-related changes in calcium buffering and inhibitory synaptic transmission in forebrain neurons [273]. Furthermore, when looking at human studies, intermittent fasting intervention has been found to ameliorate obesity, insulin resistance, dyslipidemia, hypertension, epilepsy, Alzheimer’s disease, and multiple sclerosis and improve mitochondrial organization as well as ward off inflammation [271,274,275]. Future research will need to disentangle if there are different outcomes derived from the specific fasting pattern used, the specific nutrients ingested, and total caloric intake as they relate to counteracting mitochondrial dysfunction in aging and brain-related disorders.

## 16. Physical Exercise

Physical exercise is another non-genetic intervention proven to produce long-term benefits that are multi-systematic by positively influencing multiple body systems, including muscular, vascular, nervous, immune, and endocrine systems, and also reduces all-cause mortality as well as enhancing longevity [276]. Furthermore, it has been concluded that repeated, daily exposure to a single-stress stimulus, such as exercise, improves stress resistance and immunity, increases organ function, and improves mitochondrial function by increasing the efficiency of mitochondrial biogenesis, recycling, and damage removal [277]. 

Specifically, aerobic exercise training (AET) has been shown to be effective in improving mitochondrial biogenesis, insulin sensitivity, and cardiorespiratory fitness. In older adults, AET has been found to moderately reverse mitochondrial impairment by increasing mtDNA copy number and improving oxidative enzyme function, mitochondrial transcript and protein expression, ATP production, and mitochondrial volume [278]. Resistance exercise training (RET) has been shown to have minimal effects on mitochondrial biogenesis; however, RET has been found to have marked effects on the rejuvenation of the mitochondrial transcriptome profile, enhances antioxidant enzyme activity, and reduces oxidative damage in skeletal muscle from older adults [279]. Additionally, studies have shown that acute contractile activity is actually a hormetic stress stimulus and as such can temporarily alter intracellular danger signals, such as ROS, lower the cellular energy state, and promote the release of hormones and circulatory factors. In turn, signaling pathways are activated by exercise that promote antioxidant defenses, mitochondrial biogenesis, waste recycling, and proteasome activity of the immune system [279]. For example, a recent clinical study demonstrated that completing more than 8900 steps per day may protect against age-related deficits such as cognitive decline and even slow down the progression of neurodegeneration [280]. Additional evidence indicates that walking, especially high intensity and regardless of the duration, is associated with improved episodic memory, a cognitive domain associated with Alzheimer’s disease, even when started in mid-life [281].

Alongside mitochondrial dysfunction, a multitude of groups have evaluated the efficacy of voluntary exercise in ameliorating aging and brain aging phenotypes. For example, we, and other groups, found that voluntary exercise significantly improved the premature aging phenotypes of the mtDNA mutator mouse, such as kyphosis, alopecia, decreased locomotion, and decreased motor learning, but had no major effects on lifespan [154]. Additionally, we found that exercise decreased mtDNA mutation load in skeletal muscle and rescued the proteomic profile in skeletal muscle and brain, with the majority of normalized proteins involved in mitochondrial function and nuclear-mitochondrial crosstalk. Furthermore, it has been found that voluntary exercise significantly reduced extracellular Aβ plaque load in frontal cortex from the TgCRND8 mouse model of AD [282]. Therefore, voluntary exercise may serve as a crucial tool in preventing age-related mitochondrial dysfunction as well as counteracting aging and brain aging phenotypes.

## 17. Prevention of Drug-Induced Mitochondrial Toxicity

Several drugs have been found to be able to disrupt proper mitochondrial functioning in a number of ways, such as through the inhibition of the mitochondrial ETC complexes, mitochondrial transporters, mitochondrial transcription and translation, and enzymes of the citric acid cycle, as well as through the uncoupling of ATP synthase (Complex V) [283]. Furthermore, drug-induced mitochondrial toxicity has been hypothesized to cause organ toxicity to the liver, kidney, skeletal muscle, heart, and CNS. Drug classes known to cause mitochondrial toxicity include: anti-diabetic drugs (thiazolidinediones, biguanides, fibrates), anti-depressants (serotonin antagonist and reuptake inhibitors—SARIs), pain medications (NSAIDs), cholesterol lowering drugs (statins), anti-cancer drugs (kinase inhibitors and anthracyclines), and certain antibiotics (fluoroquinolones, macrolides) [284]. Importantly, the aged population (defined as persons older than 65 years) are not only predisposed to mitochondrial toxicity by having suboptimal mitochondrial function due to age, but by also experiencing one or more of the age-related diseases mentioned above. Furthermore, the aged population are often taking multiple medications (polypharmacy), which may lead them to develop mitochondrial toxicity and dysfunction, and potentially, a brain aging disorder. Fortunately, this trend may be counteracted, and even prevented, by the administration of natural compounds or drugs that treat mitochondrial diseases and protect mitochondrial function within this population. 

## 18. Dietary Supplements 

### 18.1. Antioxidants 

Antioxidants are endogenous or exogenous substances that delay or inhibit the oxidation of a substrate, but only when present in minimal amounts. Endogenous antioxidant defenses may be enzymatic (e.g., superoxide dismutase, glutathione peroxidase [GSHPx], and catalase) or non-enzymatic (e.g., uric acid, bilirubin, thiols, glutathione (GSH), albumin, and nutritional factors, including vitamins and phenols) [285]. The most important source of endogenous antioxidants is through food, especially the phenol family, which can be found in significant amounts in berries, pomegranates, grapes, green and black tea, olives, and cocoa [286]. Phenolic compounds are considered to have beneficial biological effects on mitochondria, for example, by regulating mitochondrial biogenesis and inactivating ROS, making these compounds an attractive therapeutic strategy to combat aging and brain aging disorders [287,288]. Furthermore, endogenous antioxidant defenses are used to balance ROS production and protect the organism from oxidative stress and damage. Considering that both ROS and oxidative stress play key roles in the progression of brain aging disorders, the manipulation of these components may produce a promising treatment option in alleviating the symptoms associated with a variety of these diseases. Several compounds that present with pro-antioxidant defenses, including GSH, coenzyme Q10, vitamin E, vitamin C, and polyphenols have been reported to have mixed results [237,287]. Interestingly, it has been suggested that these antioxidants may need to be administered earlier in life to be effective [289]. Although a variety of antioxidants have been found to enhance longevity and decrease aging phenotypes within rodent models, further investigation into their underlying mechanisms and applications in humans is still necessary, with care given to include larger population samples and use randomized controlled trials (RCT), which would help control for cofounding factors, such as variations in diet and physical activity.

### 18.2. Vitamin D 

Vitamin D is a micronutrient that is metabolized into a multifunction secosteroid hormone and is essential for human health. 1,25-dihydroxyvitamin D [1,25(OH)2D], the active form of vitamin D, is generated in both the renal tubular cells and the extrarenal target tissue cells, displaying endocrine, paracrine, and autocrine functions within the body [290]. Importantly, vitamin D has been found to interact with mitochondria in several ways to enhance mitochondrial function. For example, gene activation after the interaction of 1,25(OH)2D with the vitamin D receptor (VDR) has shown importance in regulating metabolic respiration and mitochondrial integrity. Furthermore, vitamin D has been illustrated in protecting cells from elevated levels of mitochondrial respiration and the overproduction of ROS [291] as well as playing a central role in overcoming inflammation, minimizing oxidative stress, destroying invading microbes, and controlling the aging process [290]. During vitamin D deficiency, mitochondrial respiration is reduced due to a decline in the nuclear mRNA molecules and proteins that assist with mitochondrial respiration. Additionally, the production of ATP declines because of a reduction in vitamin D-dependent expression of the mitochondrial ETC, specifically in Complex I. This decline in the ETC yields an increase in the production of ROS, inducing oxidative stress and damage, and eventually, an aging phenotype [292]. Evidence from human and animal studies indicate that vitamin D deficiency increases the risk of developing accelerated brain aging with structural changes in total brain and gray matter volumes as well as functional cognitive impairments and Alzheimer’s disease [293]. Luckily, vitamin D deficiency can be corrected through loading doses of oral vitamin D or safe sun exposure. After correction, adequate maintenance doses of vitamin D are needed, and can be achieved in approximately 90% of the adult population with typical vitamin D3 supplementation, with suggested ranges of 1000 to 4000 IU/day, 10,000 IU twice a week, or 50,000 IU twice a month [294].

### 18.3. Coenzyme Q

Coenzyme Q (CoQ or ubiquinone) is a naturally occurring quinone ubiquitously found in mammals and most bacteria. In humans, the most common form is coenzyme Q_10_ (CoQ10). The primary biochemical action of CoQ is to accept electrons from Complexes I and II, thereby participating in the mitochondrial ETC. CoQ is also an important free-radical–scavenging antioxidant and is primarily located in the hydrophobic domain of the phospholipid bilayer of the IMM, but has also been found in other biological membranes as well as in plasma lipoproteins [238,295]. Recently, it has been proposed that CoQ10 supplementation may improve the symptoms associated with various mitochondrial diseases and aging phenotypes by decreasing oxidative stress and damage, improving mitochondrial bioenergetics, protecting the cell from DNA damage, and activating signaling pathways connected with aging [296]. Interestingly, using in-depth proteomics we found lower levels of CoQ10 in skeletal muscle from prematurely aging mtDNA mutator mice, which were normalized with exercise, thus suggesting that CoQ supplementation, perhaps together with exercise, could be used to treat patients with mitochondrial dysfunction [154]. Moreover, AD patients with low CoQ10 levels were found to have high serum Aβ and Aβ 40/42 ratio levels [297]. The impact of CoQ10 supplementation has been widely investigated within rodent models and has been found to improve the symptoms associated with various brain aging disorders. Oral administration of CoQ10 was found to reduce neuronal degeneration and increase longevity in toxin-induced transgenic animal models of HD and PD [298]. In transgenic mouse models of ALS, HD, and AD, CoQ10 was found to produce modest effects of neuroprotection by reducing the levels of oxidative stress in brain, reducing Aβ42 levels, decreasing β-amyloid plaque area and number, and improving overall cognition [238]. Extensive and detailed pre-clinical studies utilizing CoQ10 have strongly supported its use as a potential therapeutic agent. With that being said, an issue that these studies raise is the barrier in translating successful animal studies into application of human brain aging disorders. As a result, the therapeutic range of CoQ10 supplementation may be much higher than the doses that have been studied, especially considering that the human CNS bioavailability of CoQ10 remains unknown. Therefore, improvements of animal models and the discovery of relevant biomarkers aimed at tracking disease progression are ways in which this obstacle may be addressed [299]. 

### 18.4. Melatonin 

Melatonin or 5-methoxy-*N*-acetyltryptamine is a tryptophan derivative and was first extracted from the bovine pineal gland by Aaron Lerner in 1958 [300]. Although primarily produced in the pineal gland, extrapineal melatonin has also been found to be produced in retina, bone marrow cells, platelets, lymphocytes, skin, Harderian gland, cerebellum, and gastrointestinal tract [301]. Also known as the “hormone of darkness”, melatonin functions to regulate the circadian rhythm and sleep-wake cycle [302]. Many groups have shown that mitochondrial function impacts melatonin synthesis and in turn, melatonin provides benefits to mitochondrial function. It was found that the cytoplasm of pinealocytes is rich in mitochondria, and mitochondrial density in pinealocytes is several-fold higher than in neurons [303]. Melatonin has been shown to protect mitochondria by maintaining the mitochondrial membrane potential, inhibiting MPTP, scavenging ROS, activating UCPs, and preserving other facets of mitochondrial function [304]. Interestingly, melatonin supplementation has been shown to enhance lifespan and mitochondrial membrane potential in transgenic fruit flies overexpressing human Aβ42 [305]. Melatonin was also shown to increase mitochondrial ETC Complex I and IV activities, suggesting a strong pharmacological use of melatonin for neurodegenerative disorders [306]. Although melatonin was found to improve sleep in MCI and AD patients, the efficacy of using melatonin supplementation as a single therapeutic needs to be further investigated [307]. 

### 18.5. Herbal Medicine 

Herbal medicine is a therapeutic strategy developed long before recorded history and is utilized by countries throughout the world where treatment with medicinal plants is used in the prevention and treatment of diseases [308]. Herbal medicine has been implicated as a potential therapeutic tool in preserving, maintaining, and ameliorating mitochondrial function in aging and neurodegenerative disorders. A well-established herb used to target mitochondrial dysfunction in brain aging disorders includes red ginseng extract and is developed from steaming and drying the Araliaceae plant Ginseng root. This herb has been shown to decrease mitochondrial swelling and mitochondrial membrane potential permeability, increase mitochondrial biogenesis, and increase mitochondrial function through activation of the AMPK/PGC-1α pathway in mice [309]. Moreover, red ginseng supplementation was found to improve Aβ-induced mitochondrial toxicity, decrease Aβ deposition, gliosis, and neuronal loss, as well as improve hippocampal neurogenesis in the AD mouse model 5XFAD [310], highlighting its potential use as a therapeutic for brain aging disorders. 

## 19. Pharmacologics

The use of clinical pharmacologics to rescue, preserve, and improve mitochondrial function in aging and neurodegenerative disorders has become widespread in the last century as lifespan has significantly increased and mitochondrial dysfunction has been hypothesized as playing a key role within these pathologies. One such drug includes metformin, a medication that is used primarily to treat diabetes (type 2), but which has been indicated as a potential therapeutic for PD and AD. For example, Mor and colleagues found that treatment with metformin restored mitochondrial respiration and resultingly rescued neuronal viability through the inhibition of Complex I in *C. elegans* expressing PD-like pathology [311]. One clinical trial, MILES (Metformin in Longevity Study), discovered that metformin treatment induced tissue-specific effects on gene expression patterns associated with aging and mitochondria in adipose tissue [312]. Moreover, the currently ongoing TAME (Targeting Aging with Metformin) clinical study is examining the role of metformin as a therapeutic for aging and associated neurodegenerative diseases [313].

Another pharmacologic currently used as a treatment for type II diabetes, liraglutide, a human glucagon-like peptide 1 (GLP-1) analogue, is currently under investigation as a potential therapeutic for aging and neurodegenerative disorders [314]. For example, Vaittinen and colleagues discovered that both short- and long-term treatment with liraglutide promoted mitochondrial respiration and biogenesis and recovered TNF-induced defects in mitochondrial respiration in human adipocytes [315]. Additionally, 10-weeks of treatment was found to improve memory and decrease Aβ plaque load, synaptic loss, and neurodegeneration in APP/PS1 mice [316]. A clinical trial, ELAD, is currently ongoing and aims to investigate the effect of liraglutide treatment on cerebral glucose metabolic rate in key brain areas such as hippocampus, medial temporal lobe, and posterior cingulate in AD patients [317].

An additional pharmacologic, tricaprilin, is currently being implemented as a potential therapeutic for mild to moderate AD in humans. Tricaprilin is a semi-synthetic medium-chain triglyceride (MCT), and when administered, becomes hydrolyzed to octonoic acid and further metabolizes to ketones, which serve as an alternate energy source for the brain [318]. Interestingly, tricaprilin was not found to improve cognition in individuals with mild-to-moderate AD [319]. Nevertheless, deeper investigation into the efficacy of tricaprilin, and its ability to improve brain metabolism, is warranted.

## 20. Future Directions

Growing evidence indicates that mitochondrial dysfunction has a central role in brain aging and disease, thus treatments targeted at improving mitochondrial function may have the potential to alleviate symptoms associated with various brain aging disorders. Such treatments may include antioxidants, a regimen of exercise, caloric restriction and intermittent fasting, the prevention of mitochondrial toxicity, and more. Future efforts should work in developing new animal models that fully recapitulate the complexity of age-associated neurological disorders in order to better understand the role of mitochondrial dysfunction in these diseases and test the efficacy of existing mitochondrial-targeted therapies as well as the development of new ones. Emphasis should be put on the fact that the therapies most likely to be successful in treating brain aging disorders may have a preventive mechanism of action rather than a curative one, thus implicating that therapeutic interventions should begin at a younger age before the start of any overt symptomatology.

## Figures and Tables

**Table 1:** Mitochondrial dysfunction in brain aging disorders. Genes, mitochondrial electron transport chain (ETC) complex(es), and associated mitochondrial dysfunction in Huntington’s, Parkinson’s, and Alzheimer’s disease, with pertinent references.

Disorder	Genes Involved	Affected Mitochondrial Complex(es)	Associated Mitochondrial Dysfunction	Reference(s)
Huntington’s Disease	*HTT*	II, III, IV	Reduced functioning in mitochondrial ETC components; reduced ATP production; decreased Ca^2+^ loading capacity; altered fission/fusion dynamics; mitochondrial bioenergetic deficits; decreased mtDNA copy number; impaired mitochondrial membrane depolarization	[174,175,176,177]
Parkinson’s Disease	*PARKIN*, *PINK1*, *DJ-1*, *ATP13A2*, *SNCA*, *LRRK2*	I, II, IV	Reduced functioning in mitochondrial ETC components; decrease in mitochondrial membrane potential; decreased ATP production; increased ROS production; abnormal mitochondrial morphology; decreased mitochondrial import; decreased mtDNA copy number; increased mitophagy	[33,178,179,180,181]
Alzheimer’s Disease	*APP*, *PS1*, *PS2*, *APOE4*	IV	Reduced functioning in mitochondrial ETC components; abnormal mitochondrial morphology; decreased ATP production; increased ROS production; decreased expression and activation of mitochondrial enzymes; altered fission/fusion dynamics; decreased mitochondrial membrane potential and mtDNA copy number; abnormal mitophagy	[172,182,183,184,185,186,187]

## Data Availability

No new data were created or analyzed in this study. Data sharing is not applicable to this article.

## Abbreviations

AD	Alzheimer’s Disease
ADAF-1	apoptotic protease activating factor-1
AET	aerobic exercise training
ALP	autophagy-lysosomal pathway
ApoE	apolipoprotein E
APP	amyloid precursor protein
ATP	adenosine triphosphate (energy)
Aβ	amyloid-beta
*AβPP*	amyloid beta protein precursor
BBB	blood brain barrier
Ca^2+^	calcium
CAT	catalase
cGAMP	cyclic dinucleotide 2′3′-Cyclic GMP-AMP
cGAS	cyclic GMP-AMP synthase
CLPP	Clp protease proteolytic subunit
CoQ	coenzyme Q/ubiquinone
CoQ10	coenzyme Q_10_
CR	caloric restriction
DAMP	damage-associated molecular pattern
DOPAC	3,4-Dihydroxyphenylacetic acid
ER	endoplasmic reticulum
ETC	electron transport chain
FPR	formyl-peptide receptor
GHS	glutathione
GPx	glutathione peroxidase
GSC	germline stem cell
H^+^	protons
H_2_O	water
H_2_O_2_	hydrogen peroxide
HD	Huntington’s Disease
HO•	hydroxyl radical
HTT	huntingtin
HVA	homovanillic acid
IFN-I	type I interferon
IMM	inner mitochondrial membrane
IRF-3	interferon regulatory factor 3
KDC	ketoglutarate dehydrogenase complex
KI	knock-in
KO	knock-out
KRS	Kufor–Rakeb syndrome
LONP	lon protease homologue
MAO	monoamine oxidase
MCU	mitochondrial calcium uniporter
mDAMP	mitochondrial damage-associated molecular pattern
MFRTA	mitochondrial free-radical theory of aging
MI	myocardial infarction
MPTP	1-methyl-4-phenyl-1,2,3,6-tetrahydropyridine
mtDNA	mitochondrial DNA
MTFMT	methionyl-tRNA formyltransferase
mtROS	mitochondrial reactive oxygen species
nDNA	nuclear DNA
NFT	intracellular neurofibrillary tangle
NLR	NOD-like receptor
O_2_^•−^	superoxide radical
OMM	outer mitochondrial membrane
oxCL	oxidized cardiolipin
OXPHOS	oxidative phosphorylation
P2	purinergic
PAMP	pathogen-associated molecular pattern
PD	Parkinson’s Disease
PDC	pyruvate dehydrogenase complex
PGC-1α	Peroxisome proliferator-activated receptor-γ coactivator
PINK-1	PTEN-induced kinase-1
*PolgA*	mtDNA polymerase-γ
PRR	pattern recognition receptor
*PS1/2*	presenilin 1 or 2
pTAU	hyperphosphorylated tau
RA	rheumatoid arthritis
RET	resistance exercise training
ROS	reactive oxygen species
rRNA	ribosomal RNA
SNpc	substantia nigra pars compacta
SOD	superoxide dismutase
TCA	tricarboxylic acid cycle
TFAM	Transcription factor A mitochondrial
TLR	toll-like receptor
tRNA	transfer RNA
UPS	Ubiquitin Proteasome System
VDAC	voltage-dependent anion channel
VDR	vitamin D receptor
YAC	yeast artificial chromosome
α-Syn	alpha-synuclein
4-HNE	4-hydroxynonenal
5-Lox	5-lypoxygenase
6-OHDA	6-hydroxydopamine
8-OHdG	8-oxo-2′-deoxyguanosine
